# Moving Toward a More Comprehensive Analysis of Acceleration Profiles in Elite Youth Football

**DOI:** 10.3389/fspor.2021.802014

**Published:** 2022-01-04

**Authors:** Damian Kovacevic, George Elias, Susanne Ellens, Adam Cox, Fabio R. Serpiello

**Affiliations:** ^1^Institute for Health and Sport, Victoria University, Melbourne, VIC, Australia; ^2^Sport and Exercise Science, School of Allied Health, Human Services and Sport, La Trobe University, Melbourne, VIC, Australia

**Keywords:** acceleration, youth football, high-intensity efforts, load monitoring, injury prevention, training prescription

## Abstract

In football, having greater acceleration ability may decide the most important moments within matches. Up to now, commonly used acceleration variables have typically been investigated in isolation, with each variable suffering from unique limitations. Subsequently, any findings may provide a limited representation of what specific acceleration demands had actually occurred. Without gaining a comprehensive understanding of acceleration demands in football, it appears difficult to identify how to best monitor and maximize the long-term development of acceleration ability in footballers, all whilst doing so in a safe, sport-specific manner. Moving toward a more comprehensive analysis of acceleration profiles addresses this, as it can provide a more robust, informative understanding of the unique acceleration demands of competitive match-play. This perspective article aims to discuss the benefits of adopting a more comprehensive analysis of the acceleration demands during competitive matches for football players, by simultaneously analyzing high-intensity accelerations, repeated high acceleration ability (RHAA), and average acceleration. We discuss examples of the calculation and application of a more comprehensive acceleration profile at a team level throughout the course of an entire elite youth football season, as well as on an individual level. Monitoring acceleration profiles more comprehensively not only appears important from a training load/injury prevention perspective, but also, equips coaches and conditioning staff with the specific information necessary to develop and prescribe individualized, acceleration-emphasized training protocols that are replicable to the demands of match-play. Examples of such protocols are provided.

## Introduction

On-field football performance is determined by an interaction of technical, tactical, physical, and psychological components (Stølen et al., 2005). Among these, activities of a high intensity—including high-speed running, sprinting, and (near-) maximal acceleration efforts—are widely accepted as particularly important physical components for most outfield players (Mohr et al., 2003; Bishop et al., 2011). Whilst questions have arisen regarding the importance of some of these high-intensity activities, such as repeated-sprint ability (Buchheit et al., 2010; Carling et al., 2012), interest in (near-) maximal accelerations has progressively gained traction (Varley and Aughey, 2013). For instance, practitioners from high-level clubs around the world ranked acceleration variables as the most commonly used when monitoring players (Akenhead and Nassis, 2016). It is proposed this change in focus may be due to the theory that players are rarely afforded the time and space in football to reach absolute maximal velocities (Young et al., 2001, 2008), and are therefore constantly drawing on their ability to (near-) maximally accelerate instead.

In football, having greater acceleration ability may decide the most important moments within matches, including being first to a ball in dispute; moving into open space before an opponent to shoot, pass, or receive the ball; or, being able to press an opponent in possession of the ball, defensively (Faude et al., 2012).

Up to now, few studies in football have investigated multiple acceleration variables, simultaneously (Barberó-Álvarez et al., 2014; Barron et al., 2016; Sonderegger et al., 2016; Abbott et al., 2018a,b; Serpiello et al., 2018; Delaney et al., 2019; Martínez-Cabrera et al., 2021). Commonly used acceleration variables—including high-intensity efforts (Sonderegger et al., 2016; Abbott et al., 2018a,b; Martínez-Cabrera et al., 2021), repeated bouts (Barberó-Álvarez et al., 2014; Barron et al., 2016; Serpiello et al., 2018), and average acceleration (Delaney et al., 2018, 2019)—have typically been investigated in isolation, with each variable suffering from unique limitations. Subsequently, any findings may provide a limited representation of what specific acceleration demands had actually occurred. Without gaining a comprehensive understanding of acceleration demands in football, it appears difficult to identify how to best monitor and maximize the long-term development of acceleration ability in footballers, all whilst doing so in a safe, sport-specific manner.

To date, the classification of player acceleration data into specific movement categories (i.e., maximal or near-maximal/ “high-intensity”) has mostly been based on absolute thresholds (Bradley et al., 2010; Osgnach et al., 2010; Aughey, 2011; Castellano and Casamichana, 2013; Barron et al., 2014; Hodgson et al., 2014; Martín-García et al., 2018a,b; Oliva-Lozano et al., 2021). Although utilizing these thresholds allows for comparisons of physical performance across different cross-sectional and longitudinal studies, its primary disadvantage is that it does not account for the relative capacity of the individual player, which may be more useful in accurately determining the unique training loads exposed to each player, individually (Abt and Lovell, 2009; Abbott et al., 2018b; Martínez-Cabrera et al., 2021). This improved individualization may be important from a training prescription perspective, whereby coaches and conditioning staff can confidently replicate, overload or taper training prescription for each player, based off the unique demands they are exposed to during match-play.

The ability to repeatedly accelerate amidst short recoveries may also be important in football (Barron et al., 2016; Serpiello et al., 2018). Literature investigating repeat high-intensity accelerations in football found that repeated bouts occur frequently in matches (Barron et al., 2016; Serpiello et al., 2018), suggesting their importance and subsequent need for development. However, a limitation exists when monitoring repeated (and/or single effort) high-intensity accelerations in isolation: any accelerations that have occurred below their predetermined “high-intensity” thresholds are disregarded, efforts that would still carry their own accumulative metabolic cost (Osgnach et al., 2010; Beato and Drust, 2021). Monitoring average acceleration has been used to address this limitation, as this variable encompasses all accelerations that occur, regardless of magnitude (Delaney et al., 2018, 2019).

Using average acceleration over the course of a match (or half, or discrete period of play, etc.) provides an all-encompassing measurement of “speed change” demands (Delaney et al., 2018, 2019). Theoretically, the higher the average for a defined period, the greater the metabolic cost. As such, monitoring this variable appears important, particularly from a training load perspective [and subsequent injury prevention perspective (Cummins et al., 2013)]. Unfortunately, utilizing average acceleration as the only monitoring tool carries some limitations. Because this variable encompasses all accelerations that occur, regardless of magnitude or frequency, it may not be sensitive enough to detect the various characteristics that collectively construct acceleration demands in match-play (i.e., high- vs. low-intensity accelerations, single effort vs. repeated bouts, etc.). Without knowledge of these characteristics, it would appear difficult to develop and prescribe individualized, acceleration-emphasized training protocols replicable to the demands of match-play.

This perspective article aims to discuss the benefits of adopting a more comprehensive analysis of the acceleration demands during competitive matches for football players, by simultaneously analyzing high-intensity accelerations, repeated high acceleration ability (RHAA), and average acceleration. To exemplify this analysis, we discuss examples of the calculation and application of a more comprehensive acceleration profile at a team level throughout the course of an entire elite youth football season, as well as on an individual level.

## Materials and Methods

### Participants

Twenty-one elite youth outfield players (age 16.3 ± 0.6 years, body mass 69.9 ± 8.2 kg, height 180.2 ± 8.6 cm), from one football club, participated in this study. All players competed in the highest youth division of football in Australia, the National Premier League (NPL), in the U20 competition. As youth players have been shown to produce as many high-intensity activities as professional senior players in match-play—indicating that such activities may not be a discriminating physiological parameter between playing levels (Vigh-Larsen et al., 2018)—their participation in this perspective article was deemed appropriate. The study was conducted in accordance with the Declaration of Helsinki and obtained approval by the institutional Human Research Ethics Committee of the researcher. Prior to commencement, all players were informed of the potential risks and anticipated benefits associated with participation, and written consent was received from all respective legal guardians.

### Experimental Overview

Activity profile data was collected during 25 competitive matches of the 2018 season *via* global positioning system (GPS) devices. Once per week, players competed in a 90-min match (two 45-min halves), whilst concurrently training on their home pitch four times per week (~60 to 100-min per session). Raw GPS data was downloaded and exported using the manufacturer's software, Polar Team Pro^TM^, before being analyzed *via* a custom MatLab^TM^ script.

### Methodology

Distance, velocity, and accelerometer data were collected by commercial 10-Hz GPS devices (Polar Team Pro, Polar Electro, Kempele Finland) during 25 official league matches over a 28-week period during the 2018 NPL season. Match files were included in the analysis only when a player completed ≥75% of the total match time, resulting in 193 individual files.

Global positioning system devices were securely worn at the base of players' sternum in dedicated chest straps during data collection. Chest strap sizes were considered during each distribution prior to data collection to ensure that they be tight-fitting for each player, as unnecessary movement of GPS devices is believed to negatively affect data (Malone et al., 2017). Each player was assigned a specific device for the entirety of the data collection, in accordance with best practice GPS use in sport (Malone et al., 2017). The devices were placed in a clear outdoor space and then turned on approximately 45 min prior to administering them on the players to ensure for adequate satellite connection prior to data collection, known as a GPS “lock.” Global positioning system files were included in the analysis only when the average number of satellites and horizontal dilution of position (HDOP) obtained during the data collection was considered to be satisfactory for good GPS signal coverage based on recommendations from the manufacturer (Malone et al., 2017).

Acceleration was derived from filtered velocity data over a 0.3 s interval (Serpiello et al., 2018). Maximal acceleration capacity was calculated *via* the average 5-m acceleration of all players obtained during a 40-m sprint test (Serpiello et al., 2018), completed within a dedicated testing session, scheduled before the start of the competitive season. Maximal acceleration capacity was retested every ~6 weeks throughout the entire season, resulting in up to 5 individual 40-m sprint tests for each player. High-intensity accelerations were defined as efforts commencing above a threshold corresponding to 70% of a player's individual maximal acceleration capacity, where an effort was required to remain above the 70% threshold for at least 0.3 s to be classified as an acceleration, and was considered to be concluded when acceleration returned to or fell below 0 m·s^−2^ (Serpiello et al., 2018). Repeated high acceleration ability bouts were defined as ≥3 efforts above the 70% threshold, with ≤ 45 s recovery between each effort (Serpiello et al., 2018). Average acceleration involved taking the absolute value of all acceleration data in matches (excluding any deceleration data), and averaging it over the duration of that specific match (Delaney et al., 2018, 2019).

### Statistical Analysis

For the purpose of this perspective article, we have presented changes in each acceleration variable in relation to a smallest worthwhile change (SWC) (Hopkins et al., 2009). Smallest worthwhile change was calculated as 0.2 of the between-players, between-matches pooled standard deviation. It is recommended practitioners identify a statistical method they deem to be most appropriate for their analysis, and utilize accordingly.

## Results

**Figure 1:**
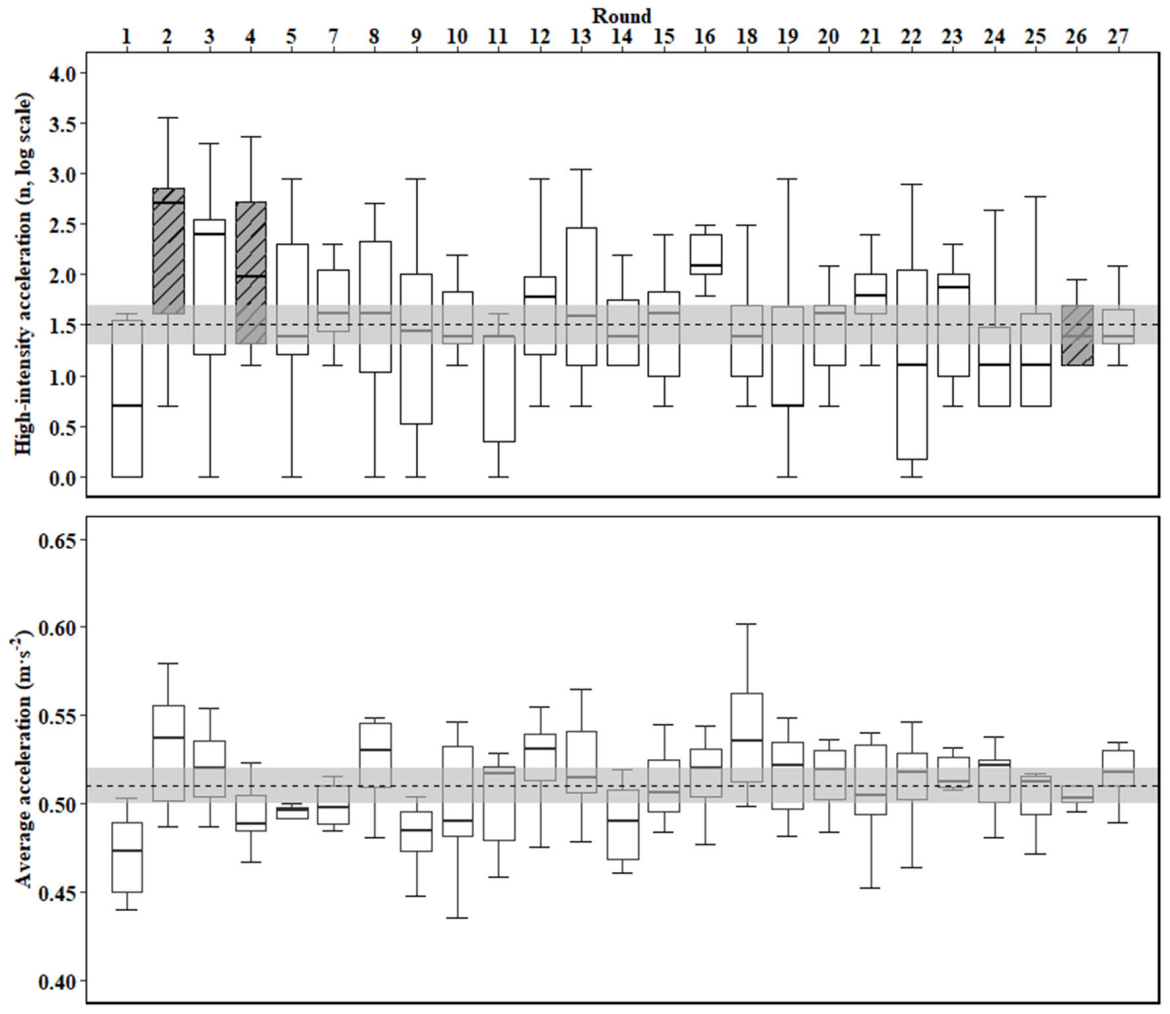
The average number of high-intensity accelerations (top panel) and average acceleration (bottom panel) during a competitive season in elite youth football (rounds 6 and 17 were both bye rounds). Dotted line (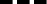) represents the team season mean; light gray band (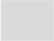) represents the smallest worthwhile change; and, patterned, dark gray boxplots (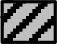) represent rounds in which at least one RHAA bout occurred from at least one player.

Team acceleration profiles are displayed in Figure 1, while an individual player profile is displayed in Table 1.

**Table 1:** The number of high-intensity accelerations, RHAA bouts, and average acceleration (m·s^−2^) in competitive matches for an elite youth footballer across his entire season (12 matches from 27 rounds).

**Round**	**HI accel (*n*)**	**RHAA bouts (*n*)**	**Ave accel (m·s^**−2**^)**
**1**	1↓	–	0.50↓
**2**	35↑	2↑	0.54↑
**3**	27↑	–	0.52
**11**	4↓	–	0.52↓
**14**	4↓	–	0.52↓
**16**	8↓	–	0.54↑
**18**	7↓	–	0.60↑
**22**	12	–	0.55↑
**23**	7↓	–	0.52
**24**	20↑	–	0.53
**25**	16	–	0.51↓
**26**	28↑	2↑	0.54
**Mean ± SD**	14.1 ± 11.1	0.3 ± 0.8	0.53 ± 0.03
**Team mean ± SD**	6.3 ± 6.0	0.0 ± 0.3	0.51 ± 0.03

*HI accel, high-intensity accelerations; n, number; RHAA, repeated high acceleration ability; Ave accel, average acceleration; ↓, output lower than smallest worthwhile change; ↑, output greater than smallest worthwhile change; SD, standard deviation*.

## Discussion

The results presented in Figure 1 and Table 1 illustrate that each acceleration variable fluctuates differently throughout the course of an entire elite youth football season. If such fluctuations are (to some extent) independent—i.e., while one variable rises, the other/s may rise or fall disproportionately—then each variable may provide unique information to coaches and conditioning staff that the others cannot. Moving toward a more comprehensive analysis of acceleration profiles addresses this, as it can provide a more robust, informative understanding of the unique acceleration demands of competitive match-play.

As an explanatory example of a more comprehensive acceleration profile on an individual level (Table 1), in round 18 the individual player that was analyzed produced the highest average acceleration for his entire season (0.60 m·s^−2^), yet produced very few high-intensity accelerations compared to his season average (7 vs. 14 ± 11 efforts). Similarly, in round 16, average acceleration was identical to rounds 2 and 26 (0.54 m·s^−2^), yet round 16 produced very few high-intensity accelerations in comparison (8 vs. 35 and 28 efforts in rounds 2 and 26, respectively). Additionally, although average acceleration was identical between these rounds, round 16 produced no RHAA bouts, whereas 2 bouts were detected in both rounds 2 and 26. Conversely, in round 3, a lower average acceleration was detected compared to round 16 (0.52 vs. 0.54 m·s^−2^), yet in round 3, the player produced notably more high-intensity accelerations in comparison (27 vs. 8 efforts).

As illustrated in the individual example above, although average acceleration is high in some rounds, this does not always correspond with equally high output from other acceleration variables (i.e., rounds 16 and 18). In these scenarios, a high occurrence of *total* accelerations can be assumed, but at magnitudes lower than 70% of maximal acceleration capacity. Efforts at lower magnitudes still carry their own accumulative metabolic cost (Osgnach et al., 2010; Beato and Drust, 2021), including subsequent increases in muscle damage and perceived muscle soreness, and decreases in neuromuscular performance (Delaney et al., 2018, 2019). As such, monitoring an all-encompassing measurement of the changes in speed demands—via average acceleration—appears important, particularly from a training load/injury prevention perspective.

Conversely, when average acceleration is low, yet high-intensity accelerations are high (i.e., round 3), large periods of low speed change demands can be assumed. However, within those periods, of the acceleration efforts that did occur, many would have been produced at high-intensity (i.e., the occurrence of a high-intensity acceleration followed by periods of low speed change demands, repeated throughout the course of the match). In this scenario, average acceleration may not have been sensitive enough to detect the various characteristics that collectively construct acceleration demands in match-play, as it encompasses all accelerations that occur, regardless of magnitude.

To demonstrate this lack of sensitivity further, we can discuss the commonly used average *sprint distance* variable as an example. A player deployed in a wide playing positions may average 120-m of sprint distance per match. Albeit an extreme illustration, this sprint distance could be comprised of 4 × 30-m efforts, or 1 × 120-m effort. Dependent on the composition of this total distance, the subsequent training prescription may be extremely different, and subsequently, demand different physical capacities be stimulated and stressed/overloaded (Tierney et al., 2018). The same can be said for acceleration demands. Without knowledge of the characteristics of accelerations demands, it would be difficult to develop and prescribe individualized, acceleration-emphasized training protocols (including “substitute top up” conditioning) that are replicable to the demands of match-play. Therefore, adopting a more comprehensive analysis of the acceleration demands during competitive matches—by analyzing multiple acceleration variables simultaneously—appears particularly beneficial here.

The low occurrence of RHAA bouts observed in this sample should be noted (0.0 ± 0.3). This may be a unique reflection of this team and, specifically, the physical demands of elite youth football in this competitive season. However, it should be acknowledged that RHAA output is invariably a product of the (arguably rigid) inclusion criteria deployed that defines a bout (≥3 efforts above the 70% threshold, with ≤ 45 s recovery between each effort). A lower threshold to determine high-intensity accelerations and/or longer maximum recoveries between each effort might have led to the detection of more RHAA bouts from the exact same sample. Regardless, unpublished internal data on competitive matches in elite female football has demonstrated that RHAA bouts do occur, particularly in important fixtures (i.e., significantly more RHAA bouts were produced vs. that of the season average in the season opener, town derby, semi-final preview, and semi-final). As such, as to prepare players for the peak acceleration demands they may be exposed to across the course of an entire competitive season, monitoring RHAA bouts still appears important.

## Conclusion

### Practical Application

Equipped with the necessary information to develop acceleration-focused training protocols informed by the demands of match-play, coaches and conditioning staff can prescribe training exercises that not only maintain average acceleration above a predetermined, match-specific value (i.e., >0.60 m·s^−2^), but also include high-intensity accelerations relative and proportionate to that of a 90-min match (i.e., if a player produces 18 efforts in a 90-min match, he/she may be required to produce 3 efforts in a 15-min match-intensity, training exercise). This prescription could be accomplished collaboratively between coaches and conditioning staff, whereby specific acceleration targets are integrated into particular football exercises.

For example, for the accrual of high-intensity accelerations, a passing exercise could include short “speed lanes,” whereby players are required to (near-) maximally accelerate from one cone to the next, after making their final pass within the exercise. Conversely, the constraints of a directional, small-sided game could be manipulated in such a way as to demand multiple high-intensity accelerations/high average acceleration from all players (i.e., all players from one team are required to be in their attacking half before being able to shoot on goal; and, after scoring, all players from the scoring team are required to (near-) maximally accelerate to their defensive goal line within ≤ 5 s for the goal to count).

Additionally, instead of collaborative prescription with coaches, conditioning staff could develop dedicated, isolated, physical prescription to achieve specific acceleration targets. For instance, for the accrual of RHAA bouts, conditioning staff could prescribe penalty box fartlek shuttles, whereby (within ≤ 45 s) players are required to (near-) maximally accelerate from the goal line to top of the nearest 6-yard goal box; gradually decelerate from the goal box to top of the nearest 18-yard penalty box; then, from the penalty box, return to the starting goal line; repeated at least twice.

### Summary of Conclusion

Commonly used acceleration variables suffer from unique limitations, and, subsequently, monitoring such variables in isolation can provide a limited representation of what specific acceleration demands have actually occurred. Without gaining a comprehensive understanding of acceleration demands in football, it appears difficult to identify how to best monitor and maximize the long-term development of acceleration ability in footballers, all whilst doing so in a safe, sport-specific manner. Moving toward a more comprehensive analysis of acceleration profiles addresses this, as simultaneously monitoring high-intensity accelerations, RHAA, and average acceleration can provide a more robust, informative understanding of these unique demands. Monitoring acceleration profiles more comprehensively not only appears important from a training load/injury prevention perspective, but also equips coaches and conditioning staff with the specific information necessary to develop and prescribe individualized, acceleration-emphasized training protocols that are replicable to the demands of match-play.

### Limitations and Methodological Considerations

A threshold corresponding to 70% of a player's individual maximal acceleration capacity was used to classify accelerations as “high-intensity,” whereas previous research has adopted different relative thresholds (Sonderegger et al., 2016; Abbott et al., 2018a,b), including thresholds corresponding to as high as 80% of maximal capacity (Serpiello et al., 2018). The 70% threshold was adopted for this perspective article as it was previously deemed appropriate for elite youth football players, specifically (Serpiello et al., 2018). It is recommended practitioners identify a relative threshold they deem to be most appropriate for their individual context, and utilize accordingly.

Furthermore, although based on previous research (Serpiello et al., 2018), the inclusion criteria deployed that defined an RHAA bout is still both arbitrary and (arguably) too rigid. Future research should look to investigate the occurrence of all acceleration efforts, irrespective of magnitude or frequency. Each effort could then be represented relatively, as a percentage of the player's individual maximal acceleration capacity (categorized into specific intensity thresholds, i.e., “high-” vs. “moderate-” vs. “low-intensity”), and frequency of efforts (or clusters) could be assessed with less rigidity.

Due to the limitations associated with the classification of continuous, time-series data into discrete thresholds and represented as a count (Varley et al., 2012; Buchheit et al., 2014; Delaney et al., 2018, 2019), such variables should be interpreted cautiously. Inferences based on “counts” (i.e., high-intensity accelerations and RHAA bouts) should rarely be compared to other contexts where different GPS devices have been used, due to differences in dwell times, filters, and smoothing techniques, etc. Along with measuring average acceleration—which is relatively stable and sensitive in comparison (Delaney et al., 2018, 2019)—practitioners could look to concurrently monitor time spent- and/or distance covered-above predefined thresholds, as these variables somewhat attenuate the limitations experienced with count data.

Practitioners could also look to monitor more of the various characteristics that collectively construct acceleration demands in match-play, including the starting velocity of each effort, peak effort value (m·s^−2^), and/or the recovery duration between efforts. Furthermore, as decelerations have been shown to have both significant metabolic *and* mechanical costs (Osgnach et al., 2010; Dalen et al., 2016), future research should look to comprehensively analyse deceleration profiles (and their equivalent characteristics), simultaneously. This additional information may further enhance the utility of acceleration (and deceleration) profiles informing match-specific training prescription, and/or overall training loads.

Football is intermittent in nature, and therefore, average acceleration is often skewed by periods of low activity (i.e., a free kick, corner, throw in, goal kick, or even periods of “slow” play). With that in mind, practitioners who wish to comprehensively analyse acceleration profiles as a means to inform their training decisions, should consider segmenting matches into shorter, discrete periods of play (i.e., first half vs. second half, discrete 15-min periods, 1–10-min peak period/s moving average/ “worst case scenario,” etc.). Segmenting matches will not only enhance the accuracy and subsequent utility of average acceleration specifically, but also, it may help establish if acceleration profiles fluctuate throughout the course of a match, and/or if individual variables within such profiles suffer from any fatiguing effects, independently.

To establish if/how much acceleration output is dictated by non-physiological components in competitive matches, future research should look to explore the relationship that match context has with the occurrence of acceleration efforts, including score line, instantaneous result (i.e., currently winning, currently losing, etc.), and strength of opposition.

## Data Availability Statement

The raw data supporting the conclusions of this article will be made available by the authors, without undue reservation.

## Ethics Statement

The studies involving human participants were reviewed and approved by Victoria University Human Research Ethics Committee. Written informed consent to participate in this study was provided by the participants' legal guardian/next of kin.

## Author Contributions

DK, GE, and FS contributed to the conceptualization, methodology and data analysis of this perspective article. DK and AC contributed to data collection. DK and SE contributed to data processing. DK contributed to the writing of all drafts. GE and FS contributed to all reviews and edits of such drafts. All authors have read and agreed to the contents of this article.

## Conflict of Interest

The authors declare that the research was conducted in the absence of any commercial or financial relationships that could be construed as a potential conflict of interest.

## Publisher's Note

All claims expressed in this article are solely those of the authors and do not necessarily represent those of their affiliated organizations, or those of the publisher, the editors and the reviewers. Any product that may be evaluated in this article, or claim that may be made by its manufacturer, is not guaranteed or endorsed by the publisher.
